# Can IRT Solve the Missing Data Problem in Test Equating?

**DOI:** 10.3389/fpsyg.2015.01956

**Published:** 2016-01-05

**Authors:** Maria Bolsinova, Gunter Maris

**Affiliations:** ^1^Department of Methodology and Statistics, Utrecht UniversityUtrecht, Netherlands; ^2^Psychometric Research Center, Dutch National Institute for Educational Measurement (Cito)Arnhem, Netherlands; ^3^Department of Psychology, University of AmsterdamAmsterdam, Netherlands

**Keywords:** item response theory, incomplete design, marginal Rasch model, missing data, non-identifiability, test equating

## Abstract

In this paper test equating is considered as a missing data problem. The unobserved responses of the reference population to the new test must be imputed to specify a new cutscore. The proportion of students from the reference population that would have failed the new exam and those having failed the reference exam are made approximately the same. We investigate whether item response theory (IRT) makes it possible to identify the distribution of these missing responses and the distribution of test scores from the observed data without parametric assumptions for the ability distribution. We show that while the score distribution is not fully identifiable, the uncertainty about the score distribution on the new test due to non-identifiability is very small. Moreover, ignoring the non-identifiability issue and assuming a normal distribution for ability may lead to bias in test equating, which we illustrate in simulated and empirical data examples.

## 1. Introduction

One of the advantages of item response theory (IRT) over classical test theory is its ability to handle incomplete designs. Among the important applications in which data are missing by design is test equating, where results of different test forms must be made comparable by accounting for the two key facts. The first is that the reference and the new tests need not be of the same difficulty, and the second is that the reference and the new populations need not have the same ability distribution (Kolen and Brennan, 2004; von Davier, 2011).

Suppose, that the same students respond both to the reference and to the new test. Assume, for the sake of the argument, that both tests are scored with a number correct score. It is clear that, if both tests represent the same underlying construct, both scores are automatically equated. The need for equating scores derives from the fact that for every student we only observe the response to either the reference or the new test. That is, it derives from the fact that there is a missing data problem.

Equating procedures are methods to overcome the missing data problem. There are many different methods for score equating with some methods based on IRT and other on classical test theory. These methods are covered in detail by, for example, Kolen and Brennan (2004), von Davier (2011), von Davier et al. (2004), Holland and Dorans (2006), and Livingston (2004). Most all equating procedures are such that all students with the same score on the reference test get the same equated score on the new test. This in contrast to both the complete data case we considered above, and more modern (multiple) imputation based techniques (Rubin, 1987).

The central question we consider in this paper is whether the distribution of the missing data (marginal or conditionally on the observed data) is in principle identifiable from the observed data. If the marginal distribution is not identifiable, neither is the conditional distribution needed to impute the missing data. Regardless of the preferred equating method, if the distribution of the missing data is not identifiable, the missing data problem can not be solved.

Suppose we take the most modest form of equating: translating the scores on the new test to a pass/fail decision (i.e., selecting a cut-score below which a student fails) consistently with the pass/fail criterion on the reference test, i.e., such the passing percentage in the reference population would be the same on the new test as it is on the reference test. To specify a new cutscore, it is sufficient to estimate the distribution of the scores of the persons from the reference population to the new test, denoted by *p*(*X*_+*mis*_)^1^. As we will show in the paper, this is not possible using an IRT model given the observed data only. Hence, solving more complicated problems of equating (obtaining a full correspondences between the scores on the two tests) is also not possible.

When IRT is used for test equating, the joint distribution of the observed data [responses of the reference population on the reference test, denoted by *p*(**X**_*obs*_)] and the missing data [responses of the reference population on new test, denoted by *p*(**X**_*mis*_)] is modeled by a marginal IRT model that consists of a conditional distribution of the data given a latent variable θ and a population distribution *f*(θ). Two elements are required to estimate the distribution of missing responses *p*(**X**_*mis*_). First, the parameters of the items from the new test and from the reference test must be placed on the common scale. Second, the ability distribution of the reference population given the observed data *f*(θ|**X**_*obs*_) must be estimated. In this paper, we have assumed that the tests are well connected through a linking design^2^ and the IRT model is correctly specified and, therefore, the first element of equating is fully satisfied. We have focused on the second element, which is usually ignored in test equating practice. The problem is that the full distribution of ability *f*(θ) is not identifiable, as has been shown by Cressie and Holland (1983). Consequently, as we show in this paper, the distribution *p*(*X*_+*mis*_) is also not identified from the observed data only. This issue is usually ignored in test equating practice, and instead a parametric distribution, usually a normal distribution, is assumed for *f*(θ). This assumption is not guaranteed to hold in practice, therefore it is important to consider to what extent the problem of inferring the distribution of missing responses can be solved without extra distributional assumptions.

We will discuss the problem of non-identifiability of *p*(*X*_+*mis*_) using the marginal Rasch model (RM) for dichotomous data, which has only one parameter in the conditional model (Rasch, 1960), as an example. The RM is chosen here for convenience; the identifiability issues are present at the level of the marginal model and are therefore not affected by the choice of a particular parametric conditional model.

In this study we investigate the extent to which the unavoidable uncertainty about the score distribution *p*(*X*_+*mis*_) that comes from non-identifiability is problematic in practice. The main purpose of this study is not to introduce a new method for test equating, but to highlight a fundamental property of marginal IRT models. This property is that in IRT equating the score distribution *p*(*X*_+*mis*_) can not be identified without making extra assumptions about the parametric shape of the ability distribution, and the practical consequences of ignoring this property.

## 2. Why IRT cannot solve the missing data problem

In this section we describe a simple model for test equating that tries (unsuccessfully) to predict missing responses from the observed data without additional distributional assumptions. The marginal RM is:

(1) $$p(X_{obs}=x)=\int_{\mathbb{R}}\prod_{i}\frac{\exp(x_{i}(\theta -\delta_{i}))}{1+\exp(\theta -\delta_{i})}f(\theta )d\theta ,$$

where **x** is a vector of dichotomous responses with *x*_*i*_ = 1 if item *i* is answered correctly and 0 otherwise; δ_*i*_ is the difficulty parameter of item *i*. There is assumed to be a population distribution *f*(θ); however, its parametric shape is not known.

Following Cressie and Holland (1983), the marginal RM in Equation (1) can be re-written as

(2) $$\begin{array}{l} p(X_{obs}=x)=\prod_{i}(\exp(-\delta_{i}){)}^{x_{i}}\int_{\mathbb{R}}(\exp(\theta ){)}^{x_{+}}\prod_{i}\frac{1}{1+\exp(\theta -\delta_{i})}\\ \;f(\theta )d\theta , \end{array}$$

where *x*_+_ is the number of items answered correctly. It can be seen that

(3) $$\begin{array}{l} f(\theta |X_{obs}=0)\propto \prod_{i}\frac{1}{1+\exp(\theta -\delta_{i})}f(\theta ), \end{array}$$

which is the posterior distribution of ability given that the responses to all items are incorrect. Therefore,

(4) $$\begin{array}{l} p(X_{obs}=x)\propto \prod_{i}(\exp(-\delta_{i}){)}^{x_{i}}E({\left(\exp(\Theta )\right)}^{x_{+}}|X_{obs}=0). \end{array}$$

To make *p*(**X**_*obs*_ = **x**) a proper density, a normalizing constant should be added. A convenient parameterisation of the marginal RM (Maris et al., 2015) is:

(5) $$\begin{array}{l} p(X_{obs}=x)=\frac{\prod_{i}b_{i}^{x_{i}}\lambda_{x_{+}}}{\sum_{s=0}^{n}\gamma_{s}(b)\lambda_{s}}, \end{array}$$

where **b** = {*b*_1_, *b*_2_, …, *b*_*n*_} is a vector of item parameters that are transformations of difficulty parameters: *b*_*i*_ = exp(−δ_*i*_); λ = {λ_0_, λ_1_, …, λ_*n*_} is a vector of population parameters, and γ_*t*_(**b**) denotes a *t*-th order elementary symmetric polynomial (Verhelst et al., 1984). The denominator ensures that the distribution integrates to 1. The model in Equation (5) is a marginal Rasch model if and only if λ is a sequence of moments of a distribution. This imposes a set of inequality constraints on the parameters (Shohat and Tamarkin, 1943):

(6) $$\begin{array}{l} \det\left[\begin{array}{cccc} \lambda_{0} & \lambda_{1} & \ldots & \lambda_{m}\\ \lambda_{1} & \lambda_{2} & \ldots & \lambda_{m+1}\\ \vdots & \vdots & \ddots & \vdots \\ \lambda_{m} & \lambda_{m+1} & \ldots & \lambda_{2m} \end{array}\right]\geq 0,m=0,1,2,\ldots \end{array}$$

and

(7) $$\begin{array}{l} \det\left[\begin{array}{cccc} \lambda_{1} & \lambda_{2} & \ldots & \lambda_{m+1}\\ \lambda_{2} & \lambda_{3} & \ldots & \lambda_{m+2}\\ \vdots & \vdots & \ddots & \vdots \\ \lambda_{m+1} & \lambda_{m+2} & \ldots & \lambda_{2m+1} \end{array}\right]\geq 0,m=0,1,2,\ldots \end{array}$$

The extended Rasch model [ERM] (Tjur, 1982; Cressie and Holland, 1983; Maris et al., 2015) does not have these restrictions.

We now apply the ERM to test equating. Let us consider the joint density of the response vectors **X**_*obs*_ and **X**_*mis*_:

(8) $$p(X_{obs}=x,X_{mis}=x^{*})=\frac{\prod_{i=1}^{n}b_{i}^{x_{i}}\prod_{j=1}^{m}d_{j}^{x_{j}^{*}}\eta_{x_{+}+x_{+}^{*}}}{\sum_{t=0}^{n+m}\gamma_{t}(b,d)\eta_{t}},$$

where **d** = {*d*_1_, …, *d*_*m*_} are the parameters of the items in the new test (analogous to **b**) and η = {η_0_, η_1_, …, η_*n*+*m*_} is a vector of (*n* + *m* + 1) population parameters corresponding to a combined test consisting of the items from both the reference and the new exams. It can be derived that the marginal distribution of the scores of the reference population on the new test is (see Appendix A, for details):

(9) $$\begin{array}{l} Pr(X_{+mis}\leq T)=\sum_{t=0}^{T}p(X_{+mis}=t)=\sum_{t=0}^{T}\frac{\gamma_{t}(d)\sum_{s=0}^{n}\gamma_{s}(b)\eta_{s+t}}{\sum_{u=0}^{n+m}\gamma_{t}(b,d)\eta_{u}}. \end{array}$$

The expression for this distribution contains parameters η, whereas the density of the observed data contains parameters λ. The parameters η and λ are related to each other as follows (see Appendix A, for details):

(10) $$\begin{array}{l} \lambda_{s}=\sum_{t\;=\;0}^{m}\gamma_{t}(d)\eta_{t+s},\forall s\in [0,n]. \end{array}$$

The parameters λ are identified from the data (up to a multiplicative constant), whereas parameters η are not; this is because in this system of (*n*+1) Equations (4) there are (*n*+*m*+1) unknowns. Therefore, having observed only data **X**_*obs*_, we cannot make direct inferences about the distribution of *X*_+*mis*_. Hence, IRT cannot solve the missing data problem.

## 3. What IRT allows us to infer about the distribution of missing responses

The conclusion at the end of the previous section does not mean that we do not know anything about the parameters η or the score distribution. The relations between λ and η impose restrictions on the values that η can take, and therefore, on the score distribution. Before considering what is and is not known about the score distribution *p*(*X*_+*mis*_), we should discussed some additional constraints for parameters η.

Along with the restriction given by the relations with the identified parameters (10), there are other restrictions that the parameters η must satisfy in order to be parameters of the ERM. First, they must be positive to ensure that all probabilities in Equation (9) are positive. To derive a second constraint, consider the probability of answering item *i* correctly given the rest score on the test:

(11) $$\begin{array}{l} \mathrm{Pr}(X_{i}=1|X_{+obs}^{\left(i\right)}+X_{+mis}=s)\\ \;=\frac{\mathrm{Pr}(X_{i}=1,X_{+obs}^{\left(i\right)}+X_{+mis}=s)}{\mathrm{Pr}(X_{+obs}^{\left(i\right)}+X_{+mis}=s)}\\ \;=\frac{b_{i}\gamma_{s}(b^{\left(i\right)},d)\eta_{s+1}}{b_{i}\gamma_{s}(b^{\left(i\right)},d)\eta_{s+1}+\gamma_{s}(b^{\left(i\right)})\eta_{s}}=\frac{b_{i}\frac{\eta_{s+1}}{\eta_{s}}}{1+b_{i}\frac{\eta_{s+1}}{\eta_{s}}},\; \end{array}$$

where **b**^(*i*)^ denotes a vector of item parameters of all items in the reference test except item *i*, and $X_{+obs}^{\left(i\right)}$ is the sum score on these items; that is, the rest score. From the measurement perspective, this probability should increase when *s* increases (Junker, 1993; Junker and Sijtsma, 2000). This ensures that all item-rest correlations are positive, so that it makes sense to score the particular set of items together as one test. For this to be true, the ratios $\frac{\eta_{s+1}}{\eta_{s}}$ must form a monotonically increasing sequence. The inequality constraint

(12) $$\begin{array}{l} \frac{\eta_{1}}{\eta_{0}}\leq \frac{\eta_{2}}{\eta_{1}}\leq \frac{\eta_{3}}{\eta_{2}}\leq \cdots \leq \frac{\eta_{n+m}}{\eta_{n+m-1}} \end{array}$$

can be specifies as a part of in the prior distribution of the population parameters (see Appendix E, for details).

An alternative motivation for using the constraints in Equation (12) is that they follow from an important feature of the marginal RM, namely that

(13) $$\begin{array}{l} \frac{\eta_{s+2}}{\eta_{s}}-{\left(\frac{\eta_{s+1}}{\eta_{s}}\right)}^{2} \end{array}$$

is the (posterior) variance of exp(θ) of a person with a score of *s* (Maris et al., 2015). The monotonicity constraints in Equation (12) follow from non-negativity of variance. Therefore, the constraints in Equation (12) are necessary but not sufficient for the parameters to satisfy the moment constraints of the marginal RM. Hence, the model we are using for equating is an ERM with the monotonicity constraints. As will be shown in the next subsection this restriction enables to reduce the uncertainty about the score distribution on the new test.

### 3.1. A simple case: *m* = 1

In this subsection we derive the uncertainty about the marginal probability of answering a new item correctly, given the observed responses to *n* items. Let us consider the simplest case in which the number of items in the new test is equal to one (*m* = 1). Because we are ignoring the effect of sampling variability on the uncertainty, we consider all identifiable parameters (**b** and λ) known.

Let λ = {λ_0_, λ_1_, …, λ_*n*_} denote the set of identifiable population parameters; η = {η_0_, η_1_, η_2_, …, η_*n*+1_} the set parameters for the combined test; and *d* the item parameter of the new item. The relations between λ and η form a system of linear equations:

(14) $$\begin{array}{l} \left(\begin{array}{c} \lambda_{0}\\ \lambda_{1}\\ \vdots \\ \lambda_{n} \end{array}\right)=\left(\begin{array}{ccccc} 1 & \text{d} & \ldots & 0 & 0\\ 0 & 1 & \ldots & 0 & 0\\ \vdots & \vdots & \ddots & \vdots & \vdots \\ 0 & 0 & \ldots & 1 & \text{d} \end{array}\right)\left(\begin{array}{c} \eta_{0}\\ \eta_{1}\\ \vdots \\ \eta_{n}\\ \eta_{n+1} \end{array}\right). \end{array}$$

This system of *n* + 1 equations does not have a unique solution because the number of unknowns (*n* + 2) is larger than the number of equations. The general solution is:

(15) $$\begin{array}{l} \left(\begin{array}{c} \eta_{0}\\ \eta_{1}\\ \vdots \\ \eta_{n+1} \end{array}\right)=\left(\begin{array}{c} k\\ \frac{\lambda_{0}}{d}-\frac{k}{d}\\ \vdots \\ \sum_{t=0}^{n}\frac{{\left(-1\right)}^{n-t}\lambda_{t}}{d^{n+1-t}}+{\left(-1\right)}^{n+1}\frac{k}{d^{n+1}} \end{array}\right), \end{array}$$

where *k* is a parameter that captures all uncertainty about η, such that the unique solution to the system of equations can be computed when *k* is known. This parameter is not completely free because η must satisfy the set of inequalities:

(16) $$\begin{array}{l} \left\{ \begin{array}{c} \eta_{s}>0,\forall s\in \left[0:\left(n+1\right)\right],\;\\ \frac{\eta_{s+1}}{\eta_{s}}\geq \frac{\eta_{s}}{\eta_{s-1}},\forall s\in \left[1:n\right].\; \end{array}\right. \end{array}$$

We are interested in the probability of answering the new item correctly, which can be written as a function of *k*:

(17) $$\begin{array}{l} \mathrm{Pr}(X_{mis}=1)=\pi_{+}(k)=\frac{d\sum_{t=0}^{n}\gamma_{t}(b)\eta_{t+1}}{d\sum_{t=0}^{n}\gamma_{t}(b)\eta_{t+1}+\sum_{t=0}^{n}\gamma_{t}(b)\eta_{t}}. \end{array}$$

Using the solutions of the system of equations, one can derive (for details, see Appendix B):

(18) $$\begin{array}{l} \pi_{+}(k)=1-\frac{k\sum_{t=0}^{n}\frac{{\left(-1\right)}^{t-1}\gamma_{t}(b)}{d^{t}}}{\sum_{t=0}^{n}\gamma_{t}(b)\lambda_{t}}+\frac{\sum_{t=1}^{n}\sum_{s=0}^{t-1}\frac{{\left(-1\right)}^{t-s}\gamma_{t}(b)\lambda_{t}}{d^{t-s}}}{\sum_{t=0}^{n}\gamma_{t}(b)\lambda_{t}}. \end{array}$$

This expression is linear in *k*. Therefore, the uncertainty about the probability of answering the new item correctly depends on the difference between the maximum and the minimum of *k*. The upper and the lower bounds for *k* can be derived from the inequalities for η in Equation (16).

From the non-negativity of the parameters η (the first set of inequalities in Equation 16), we have (see Appendix B, for details):

(19) $$\begin{array}{l} \max(0,\overset{\lfloor \frac{n+1}{2}\rfloor }{\underset{u=1}{\max}}\sum_{t=0}^{2u-1}{\left(-1\right)}^{t}\lambda_{t}d^{t})<k<\overset{\lfloor \frac{n}{2}\rfloor }{\underset{u=0}{\min}}\sum_{t=0}^{2u}{\left(-1\right)}^{t}\lambda_{t}d^{t}. \end{array}$$

Moreover, the second set of inequalities in Equation (16) leads to (see Appendix B):

(20) $$\begin{array}{l} \overset{\lfloor \frac{n-1}{2}\rfloor }{\underset{u=0}{\max}}\left(\frac{{\text{λ}}_{2u}^{2}d^{2u}}{{\text{λ}}_{2u+1}d+{\text{λ}}_{2u}}+\sum_{t=0}^{2u-1}{\left(-1\right)}^{t}{\text{λ}}_{t}d^{t}\right)\leq k\leq \overset{\lfloor \frac{n}{2}\rfloor }{\underset{u=1}{\min}}\\ \;\left(\sum_{t=0}^{2u-2}{\left(-1\right)}^{t}{\text{λ}}_{t}d^{t}-\frac{{\text{λ}}_{2u-1}^{2}d^{2u-1}}{{\text{λ}}_{2u}d+{\text{λ}}_{2u-1}}\right). \end{array}$$

Equations (19) and (20) together provide the lower and the upper bounds for *k*.

Next, we present a small example to show how the bounds on *k* change and what the uncertainty about the marginal probability of a correct response to the new item under the ERM is for different values of *n*. The item parameter *d* of this item varied from exp(−2) to exp(2), corresponding to the difficulty parameter varying from 2 to –2. We show how large the uncertainty is when only the non-negativity constraints are used, and when both the non-negativity and monotonicity constraints are used.

A data set with responses of persons sampled from a population with an ability distribution N(0, 1) to a test of six items with difficulties sampled from ln(*b*_*i*_) ~ N(0, 1) was simulated. First, only three items were taken into account, then four items, five items and, finally, all six items. We considered the identifiable parameters **b** and λ known in order to evaluate the uncertainty about π_+_ coming only from the non-identifiability of η. The identifiable parameters were fixed at their EAP-estimates obtained with a Gibbs sampler for the ERM (Maris et al., 2015), see Table 1.

**Table 1:** **Item and population parameters used in the illustrative example**.

***n***	**b**	λ
3	{1.00, 0.58, 0.41}	{1.00, 0.80, 1.16, 3.25}
4	{8.90, 1.00, 0.58, 0.41}	{1.00, 0.52, 0.45, 0.68, 1.99}
5	{8.91, 1.12, 1.00, 0.58, 0.41}	{1.00, 0.42, 0.29, 0.32, 0.60, 2.01}
6	{8.86, 1.12, 1.00, 0.85, 0.58, 0.41}	{1.00, 0.36, 0.22, 0.19, 0.27, 0.63, 2.43}

**Figure 1:**
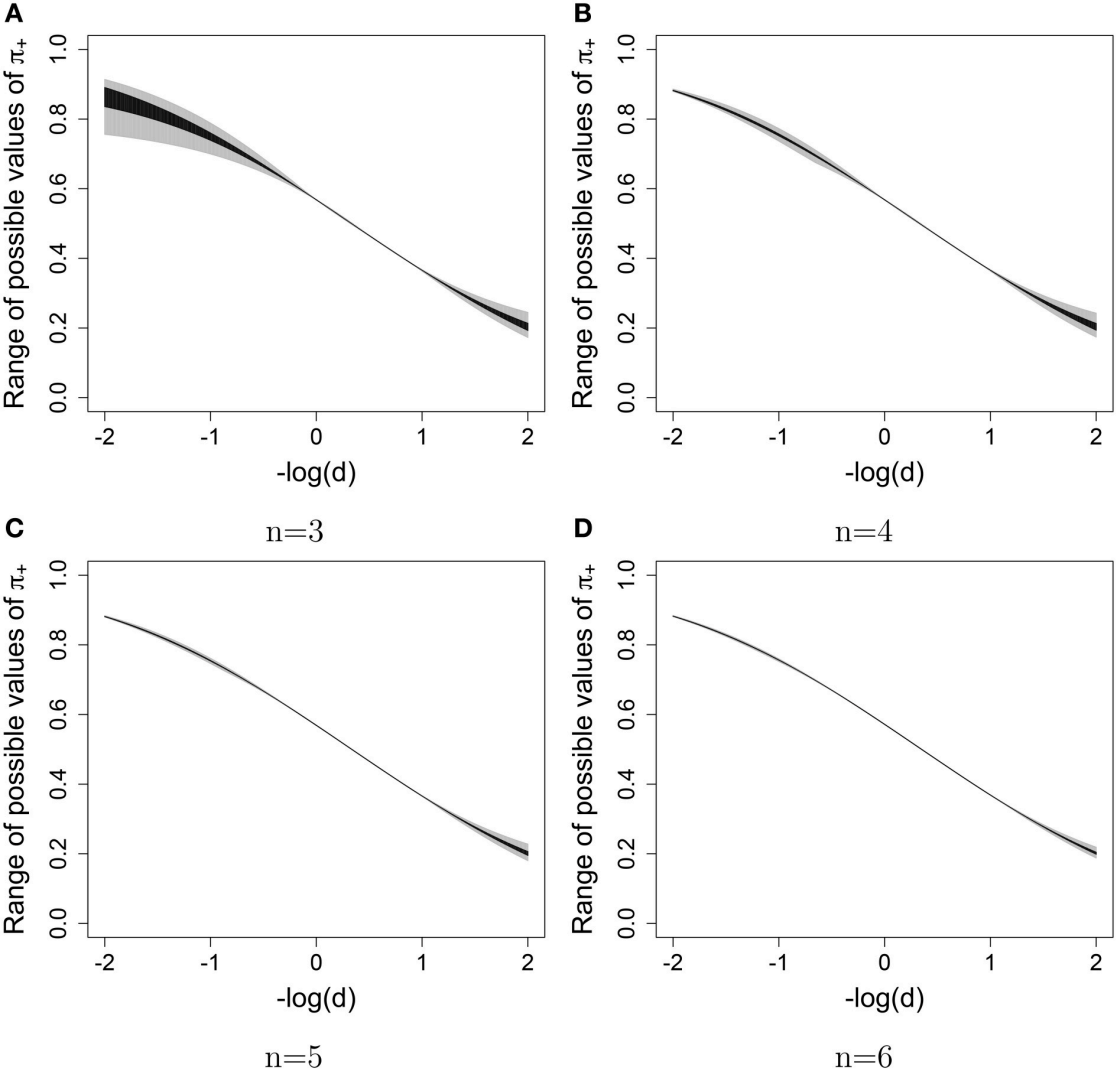
**Uncertainty about the marginal probability of answering a new item correctly (gray—without monotonicity constraints, black—with monotonicity constraints) given the difficulty of the new item (on the x-axis)**. **(A)** *n* = 3, **(B)** *n* = 4, **(C)** *n* = 5, **(D)** *n* = 6.

The possible range of values for the free parameter *k*, and therefore for the probability of interest π_+_ (given the fixed values of **b** and λ) was evaluated for different values of the difficulty of the new item. Figure 1 shows the possible ranges of values for the probability of answering the new item correctly when only the constraints in Equation (19) were used (in gray) and when the constraints in Equations (19) and (20) were used (in black).

The uncertainty about π_+_ decreases when *n* increases. For *n* = 3, the difference between the maximum and the minimum of π_+_ is for some *d* larger than 0.15 when only the constraints in Equation (19) were used and larger than 0.05 when all the constraints were used; however, when *n* = 6, the maximum discrepancy is 0.03 and 0.006 when only non-negativity constraints and all the constraints were used, respectively. Moreover, the uncertainty about π_+_ for the items with the difficulty parameter close to the items that have been answered is already very small if *n* = 3. In general, the uncertainty is larger for items with extreme difficulty^3^.

We have used this small example to explicitly show that it is not possible to compute the marginal probability of answering the new item correctly. However, there uncertainty about this probability is not large.

It is difficult to extend the analytic solution described in this section to realistic settings, with *n* and *m* being usual test lengths, because of the accumulation of error while computing the bounds for *k*. Therefore, below we present a simulation-based approach to the problem. Appendix C presents a proof of the fact that a simulation based approach is justifiable.

### 3.2. Simulated examples

This subsection provides two simulated examples to illustrate the following:

1. the size of the uncertainty about the score distribution and which part of it is due to the non-identifiability of the parameters;
2. the practical consequences of ignoring the issue of non-identifiability of *f*(θ) when the true ability distribution is not normal.

In the first example, the data were simulated according to the non-equivalent group design with three linking groups. Each group consisted of 500 persons who gave responses to 15 items from the new test and 15 items from the reference test. The relevant equating designs are described in the Appendix D. The following parameters were used: *n* = *m* = 60, *N* = *M* = 5000. Responses were simulated according to the simple RM, with person parameters sampled from N(0, 1) for the reference population, N(0.5, 0.8^2^) for the new population and N(−0.5, 2^2^), N(−0.2, 2^2^), N(−0.1, 2^2^) for the three linking groups^4^. The item difficulties (−ln *b*_*i*_) were sampled from a standard normal distribution.

**Figure 2:**
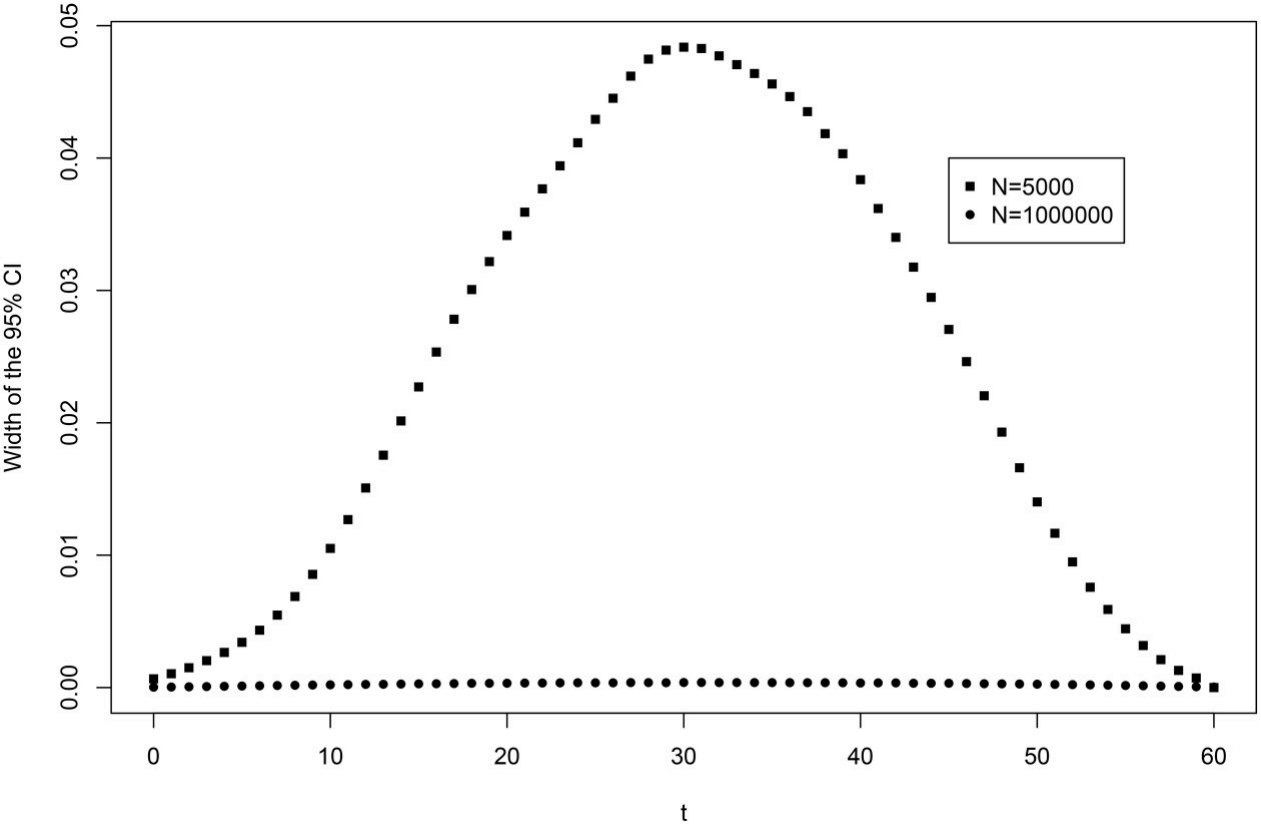
**Uncertainty about the score distribution of the reference population on the new test**.

First, the data augmented Gibbs sampler for the ERM with monotonicity constraints was used to estimate the total uncertainty about the score distribution. Second, to eliminate the uncertainty coming from the sampling variability, the new data were simulated with the same parameters but larger sample sizes (*N* = *M* = 1, 000, 000) and the algorithm was used with all the item parameters fixed at their true values. The posterior variance of the score distribution that remained was almost entirely due to the non-identifiability of the population parameters. Figure 2 presents the widths of the 95% credibility intervals of Pr(*X*_+*mis*_ ≤ *T*), ∀*T*∈[0:*m*] based on 50,000 draws from the posterior distribution after 10,000 iterations of burn-in. With a large *N* and fixed item parameters, the uncertainty about the score distribution becomes very small, not exceeding 0.002 on the probability scale.

**Figure 3:**
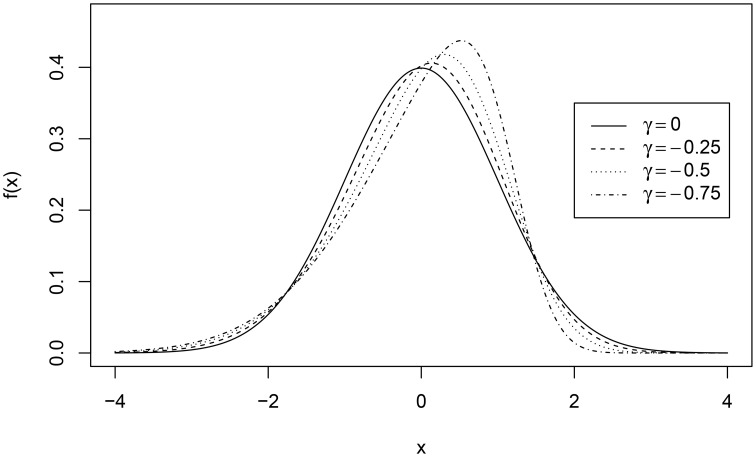
**Specification of the skewed ability distributions**.

In the second example, we compared the results of test equating using a marginal RM assuming a normal distribution of ability in the population with the results of test equating using the ERM without the normality assumption. For the R-code of the analysis and the output, see Supplementary Material. The data with different distributions of ability in the reference population were simulated. To show what happens if normality is violated, we used skew-normal distribution for ability (Azzalini, 2005). The parameters of the skew-normal distribution were chosen such that the mean was equal to 0, variance was equal to 1, and skewness was varied γ = −0.25, −0.5, −0.75. These distributions can be seen in Figure 3 (dotted lines) next to the standard normal distribution (solid line).

For each of the three degrees of skewness, we simulated the data of 5000 persons from both the reference and the new populations taking the tests, which consisted of 40 items each, connected through three linking groups consisting of 500 persons responding to 20 items (10 from the reference test and 10 from the new test). For the new population and the three linking groups, person parameters were sampled from a normal distribution [N(0.5, 0.9^2^), N(−0.5, 2^2^), N(−0.2, 2^2^), N(−0.1, 2^2^), respectively]. Item difficulties were sampled from N(0, 1). The data were simulated according to a RM.

**Figure 4:**
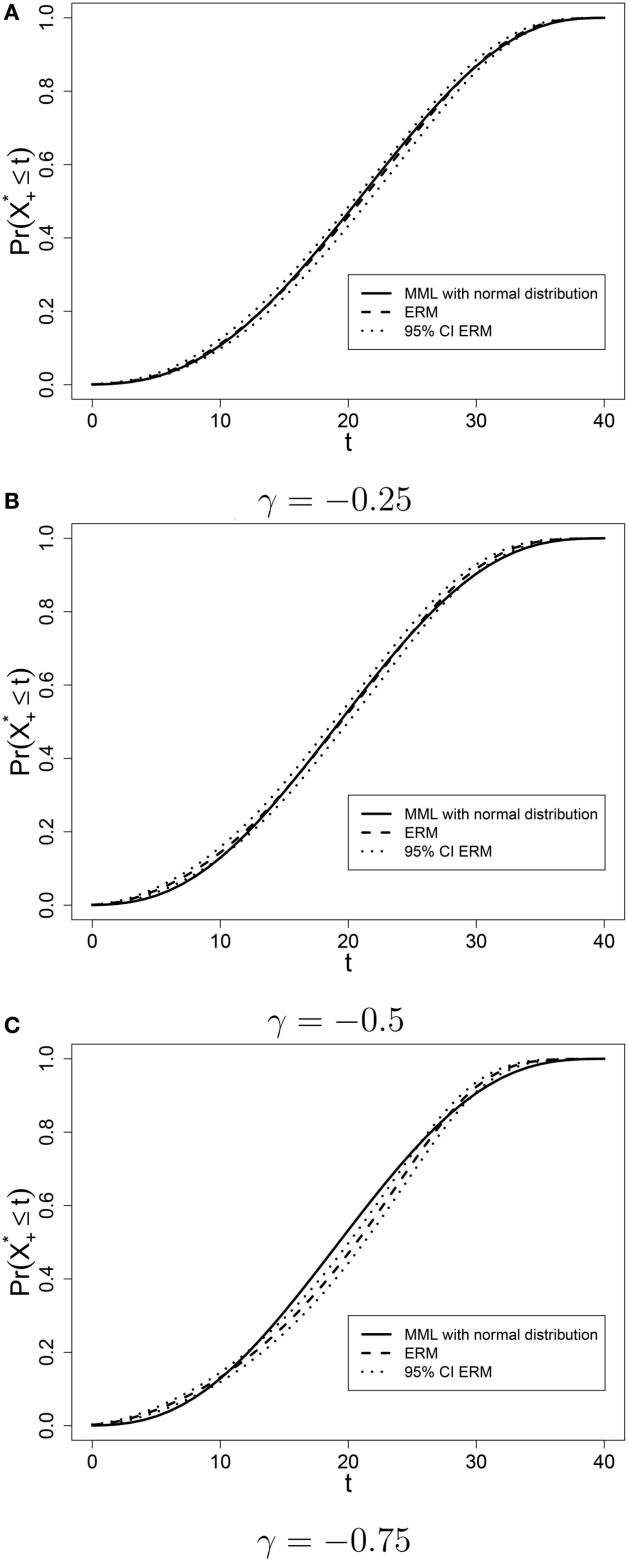
**Estimated score distributions with MML and ERM when the ability distribution in the reference population is skewed**. **(A)** γ = −0.25; **(B)** γ = −0.5; **(C)** γ = −0.75.

The score distribution Pr(*X*_+*mis*_ ≤ *T*) was estimated with marginal maximum likelihood (MML) assuming a normal distribution and with the Gibbs Sampler for the ERM. The ERM score distribution together with the 95% credibility intervals of Pr(*X*_+*mis*_ ≤ *T*) based on 50,000 draws from the posterior distribution (after 10,000 iterations of burn-in) are presented in Figure 4, together with the MML-estimate of the score distribution. The more skewed the ability distribution is, the greater the difference between equating results for the MML and ERM approaches. When γ = −0.25, the MML-estimate does not fall outside of the 95% credibility interval obtained with the ERM. When γ = −0.5, the estimate based on the normality assumption is outside the credible interval for low and high scores, but within the interval for the middle range of the scores. Finally, when γ = −0.75, the MML-estimate is also outside the credible bound in the middle range of test scores. This is the range of scores within which the cutscore is usually placed, which means that different score distributions are likely to result in different cutscores. This has consequences for the pass/fail decision for hundreds of students.

## 4. Empirical data example

Using an empirical example we show the consequences of ignoring the problem of non-identifiability of *f*(θ) and assuming a normal distribution. We do this by comparing the estimated score distributions with and without the normality assumption.

### 4.1. Method and data

**Figure 5:**
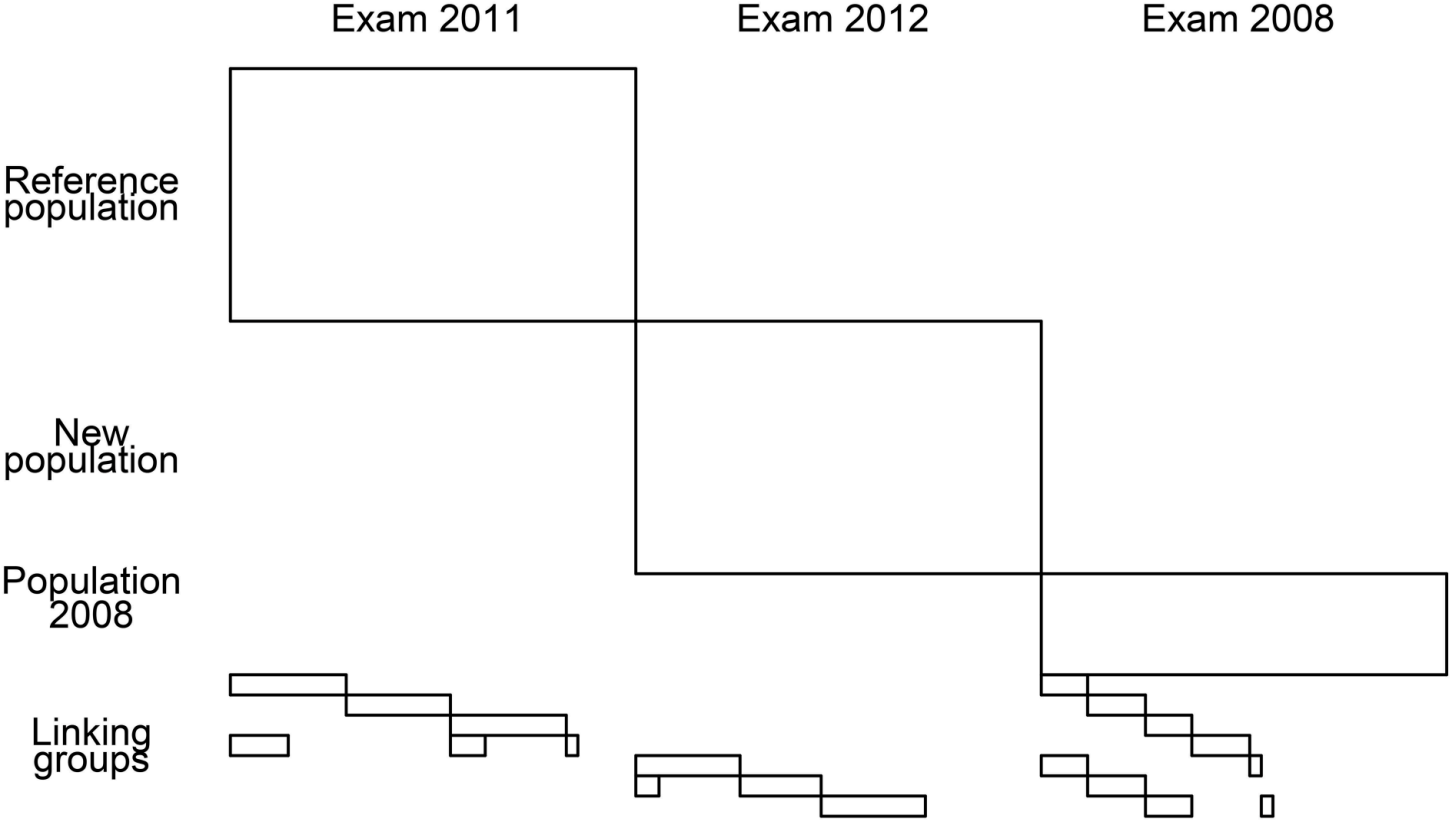
**Equating design**.

We analyzed data from the paper-and-pencil French language test for preparatory middle-level applied secondary education from examinations in 2011 and 2012. The sample sizes were 5518 for the reference exam and 5606 for the new exam. Both tests consisted of 41 items, but only dichotomous items were selected for analysis (35 and 34 in the reference and the new exams, respectively). The tests were linked through seven linking groups (with sample sizes ranging from 337 to 460) that responded to some items from either the reference test or the new test and some external anchor items (14 per group). The equating design is shown in Figure 5. There were 30 items from the reference test and 25 items from the new test answered by the linking groups. The items taken by the linking groups had been also answered by students in 2008.

First, the parameters of the ERM were estimated using the data augmented Gibbs sampler (see Appendix E). The algorithm was run for 60,000 iterations, of which the first 10,000 were discarded as a burn-in. The score distribution of the reference population on the new test was calculated at every iteration of the algorithm. Second, the marginal Rasch model with the normal distribution was fitted to the data and the MML-estimate of the score distribution was obtained. See Supplementary Material, for the data, software code of the analysis and the output.

### 4.2. Results

**Figure 6:**
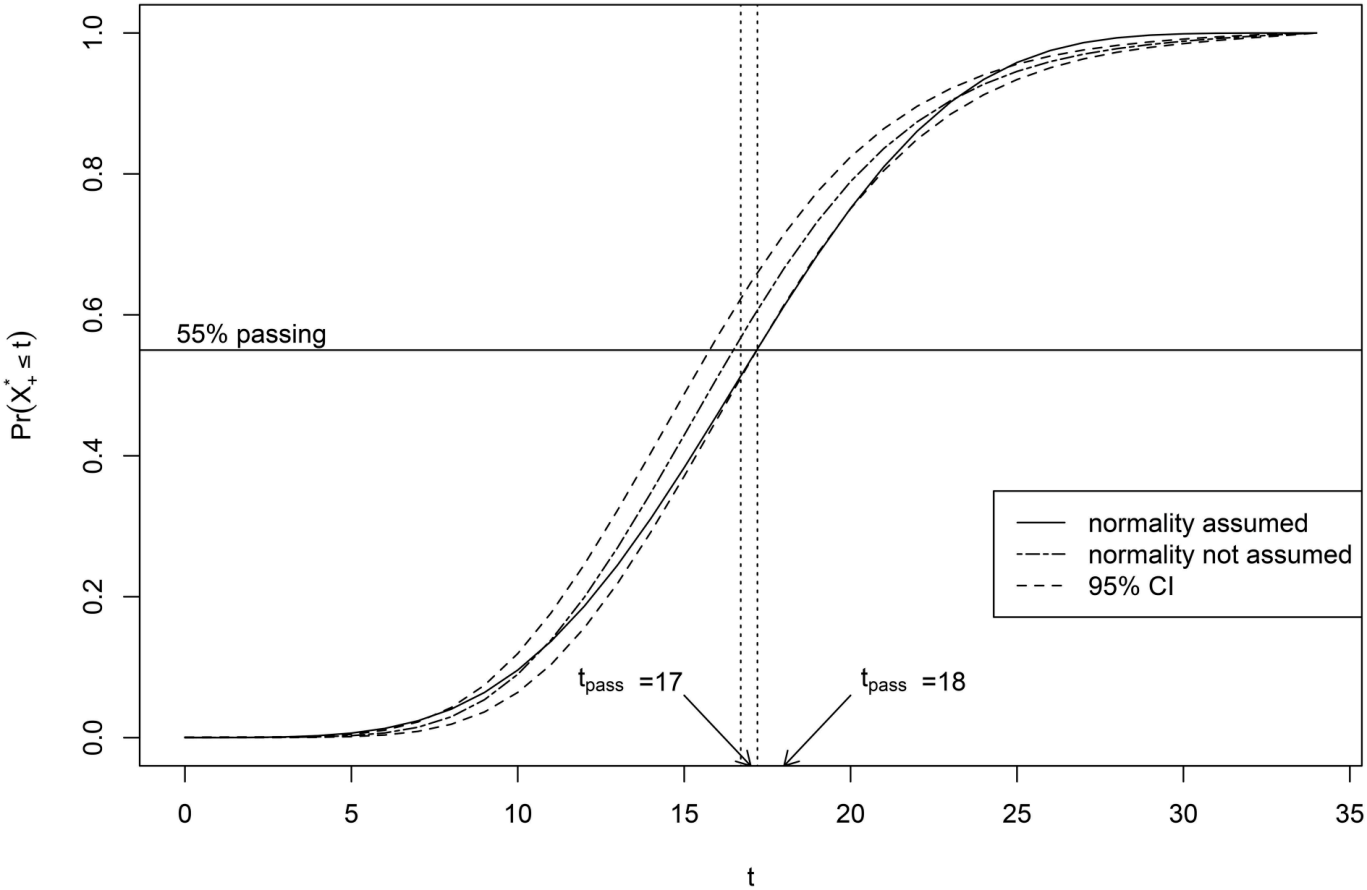
**Score distribution of the reference population on the new test: posterior mean for the ERM (dashed line) and the MML-estimate based on the assumption of the normal distribution (solid line)**.

Figure 6 shows the posterior mean of the score distribution estimated with the ERM (together with the 95% credible interval) and the MML-estimate of the the score distribution. The estimated score distributions differ and the MML-estimate is outside of the credible interval at the lower and the higher scores. The posterior mean is also different from the MML-estimate in the middle range of scores, which could have consequences for establishing the new cutscore *t*_*new*_. For example, if the desired proportion of persons from the reference population failing the new test was 55%, then the MML procedure would result in a cutscore of 17, whereas the ERM procedure would result in a cutscore of 18 as illustrated in Figure 6. The consequence of this would be that 476 students would have passed the test if a normal distribution were assumed, but would have failed if the ERM were used.

## 5. Discussion

Using a simple case, we have shown that, without the assumption of a parametric distribution, the score distribution on the new test is not identified. Knowing the difficulty parameter of the new item is not enough to predict the proportion of correct responses to this item in the population, after observing the responses to a finite set of items. When the number of items observed increases, the uncertainty about the score distribution decreases. This uncertainty tends to zero with *n* going to infinity, but is always there. Hence, IRT cannot, strictly speaking, solve the missing data problem, since it does not allow us to impute the unobserved responses of the reference population on the new test. We have investigated the degree of uncertainty about the score distribution in realistic applications. With realistic test lengths, the uncertainty coming from non-identifiability of population parameters is small enough to be ignored for practical purposes. Therefore, test equating can be done effectively without the not-fully-testable assumption of a particular parametric shape of the ability distribution, despite the non-identifiability issue.

The theoretical importance of this paper is that it has shown what one can and cannot do with respect to test equating using IRT based only on the observed data without the assumption of a parametric shape of the distribution. Although we have used the marginal RM for illustration, the issue of non-identifiability that is discussed holds in more general marginal IRT models, since the problem of the ability distribution not being identified will not go away if more parameters are added to the conditional model.

### Conflict of interest statement

The authors declare that the research was conducted in the absence of any commercial or financial relationships that could be construed as a potential conflict of interest.

## Figures and Tables

**Figure A1:**
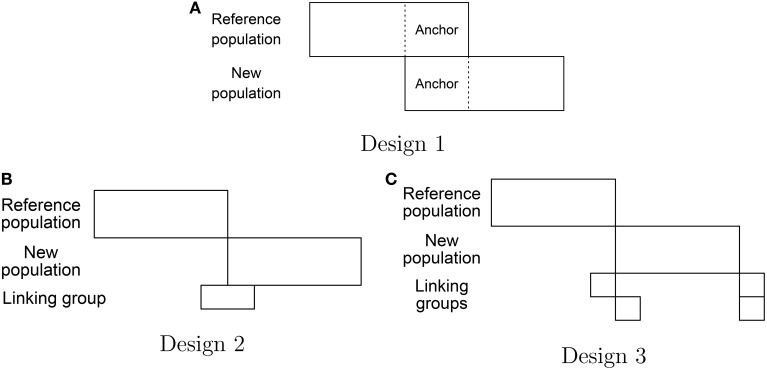
**Three non-equivalent group equating designs**. **(A)** Design 1, **(B)** Design 2, **(C)** Design 3.

## Footnotes

^1^For simplicity, we considered a situation in which the new and the reference test do not have any common items. In the general case, the missing data are responses to the items that belong to the new test but not the reference test.

^2^For a review of different linking designs see, for example, Angoff (1971), Wright and Stone (1979), Lord (1980), Petersen et al. (1989), and Kolen and Brennan (2004). Some of these linking designs are presented in Appendix D.

^3^The graphs are not symmetric because the difficulties of the items in the reference test (−ln**b**) were also not symmetric around zero.

^4^These values could be seen as matching empirical practice in the sense that the persons in the linking groups perform worse than in the examination conditions and are more heterogeneous.

## Appendix A

From Equation (8), we can derive the joint distribution of the scores on the reference test and the new test:

(A1) $$\begin{array}{c} \begin{array}{l} p(X_{+obs}=x_{+},X_{+mis}=x_{+}^{*})\\ \;\;\;\;\;\;\;\;\;\;\;\;=\frac{p(X_{obs}=x,X_{mis}=x^{*})}{p(X_{obs}=x,X_{mis}=x^{*}|X_{+obs}=x_{+},X_{+mis}=x_{+}^{*})}\\ \;\;\;\;\;\;\;\;\;\;\;\;\;=p(X_{obs}=x,X_{mis}=x^{*})/\frac{\prod_{i=1}^{n}b_{i}^{x_{i}}\prod_{j=1}^{m}d_{j}^{x_{j}^{*}}}{\gamma_{x_{+}}(b)\gamma_{x_{+}^{*}}(d)}\\ \;\;\;\;\;\;\;\;\;\;\;\;=\frac{\gamma_{x_{+}}(b)\gamma_{x_{+}^{*}}(d)\eta_{x_{+}+x_{+}^{*}}}{\sum_{t=0}^{n+m}\gamma_{t}(b,d)\eta_{t}}. \end{array} \end{array}$$

The marginal probability of obtaining a particular score on the new exam is then:

(A2) $$\begin{array}{c} \begin{array}{l} p(X_{+mis}=x_{+}^{*})=\sum_{s=0}^{n}p(X_{+obs}=s,X_{+mis}=x_{+}^{*})\\ \;=\frac{\gamma_{x_{+}^{*}}(d)\sum_{s=0}^{n}\gamma_{s}(b)\eta_{s+x_{+}^{*}}}{\sum_{u=0}^{n+m}\gamma_{u}(b,d)\eta_{u}}. \end{array} \end{array}$$

To derive the relations between the parameters λ and η, let us consider the probability of observing a particular response vector **X**_*obs*_. On the one hand, it is given in Equation (5). On the other hand, it can be presented as follows:

(A3) $$\begin{array}{c} \begin{array}{l} p(X_{obs}=x)=\sum_{t=0}^{m}p(X_{obs}=x,X_{+mis}=t)\\ =\sum_{t=0}^{m}\frac{\prod_{i=1}^{n}b_{i}^{x_{i}}\gamma_{t}(d)\eta_{x_{+}+t}}{\sum_{u=0}^{n+m}\gamma_{t}(b,d)\eta_{u}}=\frac{\prod_{i=1}^{n}b_{i}^{x_{i}}\sum_{t=0}^{m}\gamma_{t}(d)\eta_{++t}}{\sum_{s=0}^{n}\gamma_{s}(b)\left(\sum_{t=0}^{m}\gamma_{t}(d)\eta_{s+t}\right)}. \end{array} \end{array}$$

Hence,

(A4) $$\begin{array}{c} \lambda_{s}=\sum_{t=0}^{m}\gamma_{t}(d)\eta_{t+s},\forall s\in [0,n]. \end{array}$$

## Appendix B

The probability of answering the new item correctly is:

(A5) $$\begin{array}{c} \begin{array}{l} \mathrm{Pr}(X_{mis}=1)=\sum_{x}\mathrm{Pr}(X_{m}is=1,X_{obs}=x)\\ \;=\frac{d\sum_{t=0}^{n}\gamma_{t}(b)\eta_{t+1}}{d\sum_{t=0}^{n}\gamma_{t}(b)\eta_{t+1}+\sum_{t=0}^{n}\gamma_{t}(b)\eta_{t}} \end{array} \end{array}$$

Using the general solution of the system of equations in Equation (6), the two sums in this expression can be re-written as:

(A6) $$\begin{array}{l} d\sum_{t=0}^{n}\gamma_{t}(b)\eta_{t+1}=d\sum_{t=0}^{n}\left(\gamma_{t}(b)\sum_{s=0}^{t}\frac{{\left(-1\right)}^{t-s}{\text{λ}}_{s}}{d^{t+1-s}}+{\left(-1\right)}^{t}\frac{k}{d^{t+1}}\right)\\ \;=\sum_{t=0}^{n}\gamma_{t}(b)\sum_{s=0}^{t}\frac{{\left(-1\right)}^{t-s}\lambda_{s}}{d^{t-s}}+k\sum_{t=0}^{n}\gamma_{t}(b)\frac{{\left(-1\right)}^{t}}{d^{t}}\\ =\sum_{t=0}^{n}\gamma_{t}(b)\sum_{s=0}^{t-1}\frac{{\left(-1\right)}^{t-s}{\text{λ}}_{s}}{d^{t-s}}+\sum_{t=0}^{n}\gamma_{t}(b){\text{λ}}_{t}+k\sum_{t=0}^{n}\gamma_{t}(b)\frac{{\left(-1\right)}^{t}}{d^{t}} \end{array}$$

and

(A7) $$\begin{array}{l} \sum_{t=0}^{n}\gamma_{t}(b)\eta_{t}=\sum_{t=0}^{n}\left(\gamma_{t}(b)\sum_{s=0}^{t-1}\frac{{\left(-1\right)}^{t-1-s}\lambda_{s}}{d^{t-s}}+{\left(-1\right)}^{t-1}\frac{k}{d^{t}}\right)\\ \;=-\sum_{t=0}^{n}\gamma_{t}(b)\sum_{s=0}^{t-1}\frac{{\left(-1\right)}^{t-s}\lambda_{s}}{d^{t-s}}-k\sum_{t=0}^{n}\gamma_{t}(b)\frac{{\left(-1\right)}^{t}}{d^{t}}. \end{array}$$

Hence,

(A8) $$\begin{array}{l} \mathrm{Pr}(X_{mis}=1)=\frac{\begin{array}{l} \sum_{t=0}^{n}\gamma_{t}(b)\sum_{s=0}^{t-1}\frac{{\left(-1\right)}^{t-s}\lambda_{s}}{d^{t-s}}\\ +\sum_{t=0}^{n}\gamma_{t}(b){\text{λ}}_{t}+k\sum_{t=0}^{n}\gamma_{t}(b)\frac{{\left(-1\right)}^{t}}{d^{t}} \end{array}}{\sum_{t=0}^{n}\gamma_{t}(b)\lambda_{t}}=\\ =1-\frac{k\sum_{t=0}^{n}\frac{{\left(-1\right)}^{t-1}\gamma_{t}(b)}{d^{t}}}{\sum_{t=0}^{n}\gamma_{t}(b)\lambda_{t}}+\frac{\sum_{t=1}^{n}\sum_{s=0}^{t-1}\frac{{\left(-1\right)}^{t-s}\gamma_{t}(b)\lambda_{t}}{d^{t-s}}}{\sum_{t=0}^{n}\gamma_{t}(b)\lambda_{t}}.\; \end{array}$$

First, we will consider the constraints on *k*, following from the parameters η being positive:

(A9) $$\{\begin{array}{l} k=\eta_{0}>0,\\ \sum_{t=0}^{s-1}\frac{{\left(-1\right)}^{s-t+1}\lambda_{t}}{d^{s-t}}+{\left(-1\right)}^{s}\frac{k}{d^{s}}>0,\forall s\in [1:(n+1)]. \end{array}$$

For even indices $s=2u,u=1,2,\ldots ,\left\lfloor \frac{n+1}{2}\right\rfloor$, we have:

(A10) $$\begin{array}{l} \frac{k}{d^{2u+1}}>\overset{2u}{\underset{t=0}{\sum }}\frac{{\left(-1\right)}^{t}\lambda_{t}}{d^{2u+1-t}}\Leftrightarrow k>\overset{2u}{\underset{t=0}{\sum }}{\left(-1\right)}^{t}\lambda_{t}d^{t}. \end{array}$$

For odd indices $s=2u+1,u=0,1,\ldots ,\left\lfloor \frac{n}{2}\right\rfloor$, we have:

(A11) $$\begin{array}{l} \frac{k}{d^{2u}}<\overset{2u-1}{\underset{t=0}{\sum }}\frac{{\left(-1\right)}^{t}\lambda_{t}}{d^{2u-t}}\Leftrightarrow k<\overset{2u-1}{\underset{t=0}{\sum }}{\left(-1\right)}^{t}\lambda_{t}d^{t}. \end{array}$$

Second, we consider the monotonicity constraints (12): $\eta_{s+1}\eta_{s-1}>\eta_{s}^{2},\forall s\in \left[1:n\right].$ Using the the general solution of the system of equations in Equation (10), we have:

(A12) $$\begin{array}{l} \left(\sum_{t=0}^{s}\frac{{\left(-1\right)}^{s-t}\lambda_{t}}{d^{s+1-t}}+{\left(-1\right)}^{s+1}\frac{k}{d^{s+1}}\right)\\ \;\left(\sum_{t=0}^{s-2}\frac{{\left(-1\right)}^{s-t-2}\lambda_{t}}{d^{s-1-t}}+{\left(-1\right)}^{s-1}\frac{k}{d^{s-1}}\right)\\ \;>{\left(\sum_{t=0}^{s-1}\frac{{\left(-1\right)}^{s-t-1}\lambda_{t}}{d^{s-t}}+{\left(-1\right)}^{s}\frac{k}{d^{s}}\right)}^{2}. \end{array}$$

If we multiply both sides by *d*^2*s*^ and denote $S=\sum_{t=0}^{s-2}{\left(-1\right)}^{s-t}\lambda_{t}d^{t}$, then we get

(A13) $$\begin{array}{r} \left(S+\lambda_{s}d^{s}-\lambda_{s-1}d^{s-1}-{\left(-1\right)}^{s}k\right)\left(S-{\left(-1\right)}^{s}k\right)\\ >{\left(-S+\lambda_{s-1}d^{s-1}+{\left(-1\right)}^{s}k\right)}^{2}. \end{array}$$

When multiplying the elements on the left side and taking a square on the right side, most of the element on the both sides are the same, hence they cancel out, and the remaining inequality is:

(A14) $$\begin{array}{l} (S-{\left(-1\right)}^{s}k)\lambda_{s}d^{s}>-(S-{\left(-1\right)}^{s}k)\lambda_{s-1}d^{s-1}+{\left(\lambda_{s-1}d^{s-1}\right)}^{2}\Leftrightarrow \\ \;S-{\left(-1\right)}^{s}k>\frac{\lambda_{s-1}^{2}d^{s-1}}{\lambda_{s}d+\lambda_{s-1}} \end{array}$$

For even indices $s=2u,u=1,2,\ldots ,\left\lfloor \frac{n}{2}\right\rfloor$, we have:

(A15) $$\begin{array}{l} k<\overset{2u-2}{\underset{t=0}{\sum }}{\left(-1\right)}^{t}\lambda_{t}d^{t}-\frac{\lambda_{2u-1}^{2}d^{2u-1}}{\lambda_{2u}d+\lambda_{2u-1}}. \end{array}$$

For odd indices $s=2u+1,u=0,1,\ldots ,\left\lfloor \frac{n-1}{2}\right\rfloor$, we have:

(A16) $$\begin{array}{l} k>\frac{\lambda_{2u}^{2}d^{2u}}{\lambda_{2u+1}d+\lambda_{2u}}+\overset{2u-1}{\underset{t=0}{\sum }}{\left(-1\right)}^{t}\lambda_{t}d^{t}. \end{array}$$

## Appendix C

For a simulation approach (such as a Gibbs Sampler) to be applicable, we have to show that the solutions of the system of Equation (10) and inequalities (16) constitute a convex and bounded set, which ensures that the sampler can easily cover the full subspace of possible values of the non-identified parameters. All coefficients in the system of equations are positive, so are the parameters λ, and therefore each of the parameters η_*s*_ is bounded:

(A17) $$\begin{array}{l} 0<\eta_{s}<\overset{\min\left(s,n\right)}{\underset{t=\max\left(0,\;s-m\right)}{\min}}\frac{\lambda_{t}}{\gamma_{s-t}\left(b\right)},\forall s\in \left[0:\left(n+m\right)\right]. \end{array}$$

For every *s* ∈ [1 : (*n* + *m* − 1)] the solutions of the following set of inequalities:

(A18) $$\{\begin{array}{l} \frac{\eta_{s+1}}{\eta_{s}}\geq \frac{\eta_{s}}{\eta_{s-1}}\\ \eta_{s-1}>0\\ \eta_{s}>0\\ \eta_{s+1}>0 \end{array}$$

form a convex set. The interaction of convex sets from each *s* is itself a convex set. The intersection of the set formed by solutions of all inequalities and the set formed by the system of linear equations (which is always a convex set) is also a convex set. Therefore, all possible values of η constitute a convex set, and for each individual parameter there is only one range of possible values. Although the parameters are not identified, it is still possible to sample from their joint posterior distribution. The data augmented Gibbs Sampler for test equating with the ERM which is an extension of the algorithm of Maris et al. (2015) was developed for this. The details of our algorithm can be found in the Appendix E.

## Appendix D

### Non-equivalent group equating designs

The most simple non-equivalent group design one is the anchor-item design, in which both the reference and the new tests include a common set of items. The second design is a post-equating design, in which the link between the two tests is established through the data collected in the so called linking groups, answering some items from the reference test together with some items from the new test. The third design is a variation of the post-equating design, in which persons from some of the linking groups answer the items from the reference test and some other items from the item bank, while persons from the other linking groups answer the items from the new test and the same items from the item bank. The items that do not belong to either the reference test or the new test might also be taken by students from some historic population in one of the previous years. The simplest forms of these designs are visualized in Figure A1.

Let us by **Y** denote the *M*×*m* data matrix with responses of a sample of persons from the new population to the new test, by κ denote the *m*+1 identified population parameters of the new population, by {*r*} the set of items in the reference test and by {*c*} the set of items in the new test. If the design includes linking groups, then **Z** is the data coming from the equating groups with *K*^(*g*)^ and *k*^(*g*)^ being the number of persons in the *g*-th linking group and the number of items answered by them; {*e*^(*g*)^} denotes the set of items answered by the *g*-th linking group and τ^(*g*)^ are the population parameters of this linking group. Then the density of the observed data is:

(A19) $$\begin{array}{l} f(X_{obs},Y,Z)=\frac{\prod_{i}b_{i}^{u_{i}}\prod_{s=0}^{n}{\left(\sum_{t=0}^{\left|c/r\right|}\gamma_{t}(b_{c/r})\eta_{t+s}\right)}^{N_{s}}\prod_{s=0}^{m}\kappa_{s}^{M_{s}}}{{\left(\sum_{t=0}^{\left|r\cup c\right|}\gamma_{t}(b_{r\cup c})\eta_{t}\right)}^{N}{\left(\sum_{t=0}^{m}\gamma_{t}(b_{c})\kappa_{t}\right)}^{M}}\\ \;\prod_{g}\frac{\prod_{s=0}^{k^{\left(g\right)}}{\left(\tau_{s}^{\left(g\right)}\right)}^{K_{s}^{\left(g\right)}}}{{\left(\sum_{t=0}^{k^{\left(g\right)}}\gamma_{t}\left(b_{e^{\left(g\right)}}\right)\tau_{t}^{\left(g\right)}\right)}^{K^{\left(g\right)}}}, \end{array}$$

where *u*_*i*_ is the total number of correct responses to item *i* by all students which answered this item, *N*_*s*_, *M*_*s*_, and $K_{s}^{\left(g\right)}$ are the number of persons from the reference population, new population and the *g*-th linking group, respectively, that gave exactly *s* correct responses to the items in the corresponding tests.

The score distribution of the reference population on the new test depends on the population parameters η and the item parameters **b**:

(A20) $$\begin{array}{l} p\left(X_{+mis}\leq T\right)=\overset{T}{\underset{t=0}{\sum }}\frac{\gamma_{t}\left(b_{c∕r}\right)\left(\sum_{s=0}^{n}\gamma_{s}\left(b_{r∕c}\right)\eta_{s+y}\right)}{\overset{\left|r\cup c\right|}{\underset{u=0}{\sum }}\gamma_{u}\left(b\right)\eta_{u}} \end{array}$$

To make inferences about this distribution we obtain samples from the posterior distribution *p*(η, **b**|… ). This is done using a data augmented Gibbs sampler.

## Appendix E

We describe here how the samples from the joint posterior distribution

(A21) $$\begin{array}{l} p\left(\eta ,b|\ldots \right) \end{array}$$

can be obtained using a Markov chain Monte Carlo algorithm. We describe it for the post-equating non-equivalent groups design (see Figure A1B) with *G* linking groups. The density of the data given this equating design is given in Equation (A19) in Appendix D. The algorithm can be easily altered for the different kinds of non-equivalent group designs.

### Data augmented gibbs sampler

For computational convenience, instead of parameters η, we use a different parametrization with the ratios of the consecutive parameters $\frac{\eta_{s+1}}{\eta_{s}}$. To place the parameters on the scale common in IRT we consider logarithms of these ratios:

(A22) $$\begin{array}{l} p_{s}=\ln\left(\frac{\eta_{s+1}}{\eta_{s}}\right),\forall s\in \left[0:n+m-1\right]. \end{array}$$

We use a prior which in addition to the monotonicity constraint (5) has a lower and an upper bound for the parameters:

(A23) $$\begin{array}{l} p\left(p\right)\propto \overset{n+m-1}{\underset{s=0}{\prod }}I_{\left[p_{s-1},p_{s+1}\right]}\left(p_{s}\right), \end{array}$$

where *p*_−1_ = −100 and *p*_*n*+*m*_ = 100. This is a reasonable constraint, since it follows from the Dutch identity (Holland, 1990) that

(A24) $$\begin{array}{l} p_{s}=\ln\left(ℰ\left(\exp\left(\Theta \right)|X_{+obs}+X_{+mis}=s\right)\right). \end{array}$$

A priori, item and population parameters are independent. For item parameters we choose a uniform prior for difficulty parameters −ln(*b*_*i*_), which is $p\left(b_{i}\right)\propto \frac{1}{b_{i}}$.

After the re-parametrization, the density of the observed data is:

(A25) $$\begin{array}{l} f(X_{obs},Y,Z^{\left(1\right)},\ldots ,Z^{\left(G\right)})=\prod_{g=1}^{G}\;\\ \;\frac{\prod_{i\in \{r\cap e_{g}\}}b_{i}^{x_{+i}+z_{+i}^{\left(g\right)}}\prod_{i\in \{c\cap e_{g}\}}b_{i}^{y_{+i}+z_{+i}^{\left(g\right)}}\prod_{s=1}^{k^{\left(g\right)}-1}\exp{\left(r_{s}^{\left(g\right)}\right)}^{\sum_{j>s}K_{j}^{\left(g\right)}}}{{\left(1+\sum_{t=1}^{k^{\left(g\right)}}\gamma_{t}(b_{e_{g}})\prod_{j<t}\exp(r_{j}^{\left(g\right)})\right)}^{K^{\left(g\right)}}}\times \\ \frac{\begin{array}{l} \prod_{i\in \{r/e\}}b_{i}^{x_{+i}}\prod_{i\in \{c/e\}}b_{i}^{y_{+i}}\prod_{s=0}^{n}{\left(1+\sum_{t=1}^{m}\gamma_{t}(b_{c})\prod_{j<t}\exp(p_{j})\right)}^{N_{s}}\\ \;\prod_{s=0}^{m-1}\exp{\left(q_{s}\right)}^{\sum_{j>s}M_{j}} \end{array}}{{\left(1+\sum_{t=1}^{n+m}\gamma_{t}(b)\prod_{j<t}\exp(p_{j})\right)}^{N}{\left(1+\sum_{t=1}^{m}\gamma_{t}(b_{c})\prod_{j<t}\exp(q_{j})\right)}^{M}}, \end{array}$$

where **p**, **q**, **r**^(1)^, …, **r**^(*G*)^ are the population parameters of the reference population, the new population and *G* linking groups, respectively.

Although, we are interested only in the parameters **b**_*c*_ and **p**, the other parameters ($b_{r},q,r^{\left(1\right)},\ldots ,r^{\left(G\right)}$) are also sampled as nuisance parameters. Moreover, to make the full conditional posterior distribution of *p*_*s*_ tractable, at every iteration we will sample augmented data **x**^*^: responses of persons from the reference group to the items of the new test (Tanner and Wong, 1987; Zeger and Karim, 1991). This amounts to sampling from the joint posterior:

(A26) $$\begin{array}{l} p\left(p,q,r^{\left(1\right)},\ldots ,r^{\left(G\right)},b,x^{*}|X_{obs},{Y,Z}^{\left(1\right)},\ldots ,Z^{\left(G\right)}\right). \end{array}$$

A Gibbs sampler is used, i.e., all parameters are subsequently sampled from their full conditional distributions given the new values of all other parameters (Geman and Geman, 1984; Casella and George, 1992). After starting from the initial values (1 for all item parameters, and the population parameters equally distanced from –3 to 3), the algorithm goes through the following steps:

**Step 1**. Sample the augmented data **x**^*^.

For every person *j*∈[1:*N*], sample a vector of responses $x_{j}^{*}$ from its full conditional posterior $p\left(x_{j}^{*}|\ldots \;\right)$, which is factored in the following way:

(A27) $$\begin{array}{l} p(x_{j}^{*}|\ldots )=p(x_{j+}^{*}|x_{j+},b_{c},p)p(x_{j}^{*}|x_{j+}^{*},b_{c})\\ \;=p(x_{j+}^{*}|x_{j+},b_{c},p)\\ \;\times p(x_{j,1}^{*}|x_{j+}^{*},b_{c})p(x_{j,2}^{*}|x_{j,1},x_{j+}^{*},b_{c})\ldots \\ \;p(x_{j,m}^{*}|x_{j,1}^{*},\ldots ,x_{j,m-1}^{*},x_{j+}^{*},b_{c}), \end{array}$$

where *x*_*j*+_ is the sumscore of person *j*. First, sample $x_{j+}^{*}$ from the categorical distribution with probabilities

(A28) $$\mathrm{Pr}(x_{j+}^{*}=s|x_{j+},p,b_{c})=\frac{\gamma_{s}(b_{c})\prod_{u<(x_{j+}+s)}\exp(p_{u})}{1+\sum_{t=1}^{m}\gamma_{t}(b_{c})\prod_{u<(x_{p+}+t)}\exp(p_{u})}.$$

And then for every item *i*∈[1:*m*] sample $x_{j,i}^{*}$ from a Bernoulli distribution with probability:

(A29) $$\mathrm{Pr}(x_{j,i}^{*}=1|x_{j+}^{*},x_{j,s<i}^{*},b_{c})=\frac{b_{i}\gamma_{x_{j+}^{*}-\sum_{s=0}^{i-1}x_{j,s}^{*}-1}(b_{i+1},\ldots ,b_{m})}{\gamma_{x_{j+}^{*}-\sum_{s=0}^{i-1}x_{j,s}^{*}}(b_{i},b_{i+1}\ldots ,b_{m})}$$

**Step 2**. Sample from the full conditional posterior of the distribution of the item parameters.

For every *i*∈{*r*∕*e*}, sample *b*_*i*_ from its full conditional posterior:

(A30) $$\begin{array}{l} p\left(b_{i}|\ldots \;\right)\propto \frac{b_{i}^{x_{+i}-1}}{{\left(1+cb_{i}\right)}^{N}}, \end{array}$$

where $c=\frac{\overset{n+m}{\underset{t=1}{\sum }}\gamma_{s-1}\left(b^{\left(i\right)}\right)\prod_{j<t}\exp\left(p_{j}\right)}{\overset{n+m-1}{\underset{t=0}{\sum }}\gamma_{s}\left(b^{\left(i\right)}\right)\prod_{j<t}\exp\left(p_{j}\right)}$. This is an scaled beta-prime distribution, to sample from which first sample $y=\frac{cb_{i}}{1+cb_{i}}$ from B(*x*_+*i*_, *N*−*x*_+*i*_), and then transform it: $b_{i}=\frac{1}{c}\frac{y}{1-y}$.

For every *g*∈[1:*G*], for every *i*∈{*r*∩*e*_*g*_}, sample *b*_*i*_ from its full conditional posterior:

(A31) $$\begin{array}{l} p\left(b_{i}|\ldots \;\right)\propto \frac{b_{i}^{x_{+i}+z_{+i}^{\left(g\right)}-1}}{{\left(1+c_{1}b_{i}\right)}^{N}{\left(1+c_{2}b_{i}\right)}^{K^{\left(g\right)}}}, \end{array}$$

where $c_{1}=\frac{\overset{n+m}{\underset{t=1}{\sum }}\gamma_{s-1}\left(b^{\left(i\right)}\right)\prod_{j<t}\exp\left(p_{j}\right)}{\overset{n+m-1}{\underset{t=0}{\sum }}\gamma_{s}\left(b^{\left(i\right)}\right)\prod_{j<t}\exp\left(p_{j}\right)}$ and $c_{2}=\frac{\overset{k^{g}}{\underset{t=1}{\sum }}\gamma_{s-1}\left(b_{e_{g}}^{\left(i\right)}\right)\prod_{j<t}\exp\left(r_{j}^{\left(g\right)}\right)}{\overset{k^{\left(g\right)}-1}{\underset{t=0}{\sum }}\gamma_{s}\left(b_{e_{g}}^{\left(i\right)}\right)\prod_{j<t}\exp\left(r_{j}^{\left(g\right)}\right)}$. Unlike the full conditional of the item parameters of the items taken by persons from only one population, this distribution is not easy to sample from directly. It is more convenient to sample from the distribution of β_*i*_ = −ln(*b*_*i*_) using a Metropolis-Hasting algorithm (Metropolis et al., 1953). We use $N\left(-\ln\left(b_{i}\right),\tau^{2}=0.01\right)$ as a proposal density with *b*_*i*_ being the current value of the parameter.

For every *i*∈{*c*∕*e*}, sample *b*_*i*_ from its full conditional posterior analogously to sampling *b*_*i*_, *i*∈{*r*∩*e*_*g*_}, because these items are not only taken by the new population, but responses to these items by the reference population are imputed.

For every *g*∈[1:*G*], for every *i*∈{*c*∩*e*_*g*_} sample *b*_*i*_ from its full conditional posterior:

(A32) $$\begin{array}{l} p\left(b_{i}|\ldots \;\right)\propto \frac{b_{i}^{y_{+i}+z_{+i}^{\left(g\right)}+x_{+i}^{*}-1}}{{\left(1+c_{1}b_{i}\right)}^{N}{\left(1+c_{2}b_{i}\right)}^{K^{\left(g\right)}}{\left(1+c_{3}b_{i}\right)}^{M}}, \end{array}$$

where $c_{3}=\frac{\overset{m}{\underset{t=1}{\sum }}\gamma_{s-1}\left(b_{c}^{\left(i\right)}\right)\prod_{j<t}\exp\left(q_{j}\right)}{\overset{m-1}{\underset{t=0}{\sum }}\gamma_{s}\left(b_{c}^{\left(i\right)}\right)\prod_{j<t}\exp\left(q_{j}\right)}$. Use the same Metropolis-Hastings algorithm as for the items, taken by two populations. If the equating design specifies more than 3 populations taking some of the items, then the full conditional posteriors of those items can be extended accordingly.

**Step 3**. Sample the population parameters.

For every *s*∈[0:(*n*+*m*−1)], sample *p*_*s*_ from its full conditional posterior:

(A33) $$p(p_{s}|\ldots )\propto \frac{\exp{\left(p_{s}\right)}^{\sum_{j>s}N_{s}^{*}}}{{\left(1+c\exp(p_{s})\right)}^{N}}{ℐ}_{\left[p_{s-1},p_{s+1}\right]}(p_{s}),$$

where $c=\frac{\sum_{t=s+1}^{n}\gamma_{t}(b)\prod_{j\neq s,j=0}^{t-1}\exp(p_{j})}{1+\sum_{t=1}^{s}\gamma_{t}(b)\prod_{j=0}^{t-1}\exp(p_{j})}.$ To sample from this distribution, we first sample $y=\frac{c\exp\left(p_{s}\right)}{1+c\exp\left(p_{s}\right)}$ from the truncated beta distribution

(A34) $$f(y)\propto y^{\sum_{j>s}N_{s}^{*}-1}{\left(1-y\right)}^{N-\sum_{j>s}N_{s}^{*}-1}{ℐ}_{\left[a_{1},a_{2}\right]}(y),$$

where $a_{1}=\frac{c\exp\left(p_{s-1}\right)}{1+c\exp\left(p_{s-1}\right)}$ and $a_{2}=\frac{c\exp\left(p_{s+1}\right)}{1+c\exp\left(p_{s+1}\right)}$, using rejection sampling with *U*(*a*_1_, *a*_2_) as a proposal distribution, and then transform it: $p_{s}=\ln\left(\frac{1}{c}\frac{y}{1-y}\right).$

For every *s*∈[0:(*m*−1)], sample *q*_*s*_ from its full conditional posterior analogously to sampling *p*_*s*_. For every *g*∈[1:*G*], for every *s*∈[0:*k*^(*g*)^−1], sample $r_{s}^{\left(g\right)}$ form its full conditional posterior analogously to sampling *p*_*s*_.

At every iteration of the Gibbs sampler, we compute the expected score distribution for the reference population on the new exam *Pr*(*X*_+*mis*_ ≤ *T*).
